# Sarcopenia and motoric cognitive risk syndrome: a moderated mediation model

**DOI:** 10.1186/s12877-022-02802-4

**Published:** 2022-02-19

**Authors:** Ting Zhang, Yunyun Zhang, Ziyan Lv, Jie Xiang

**Affiliations:** 1 Department of Rehabilitation, The Affiliated Hospital of Xuzhou Medical University, Xuzhou, 221002 Jiangsu China; 2 Medical Technology School, Xuzhou Medical University, Xuzhou, 221004 Jiangsu China; 3 The Second School of Clinical Medicine, Xuzhou Medical University, Xuzhou, 221004 Jiangsu China

**Keywords:** Sarcopenia, Motoric Cognitive Risk syndrome, Apathy, Physical activity, Moderated mediation

## Abstract

**Background:**

Sarcopenia has been identified as a risk factor for cognitive impairment, and motoric cognitive risk syndrome (MCR) is a recently defined pre-dementia syndrome. It is not known whether they are related. We aimed to investigate the association and potential pathways between sarcopenia and MCR in the community elderly by establishing a moderated mediation model.

**Methods:**

846 community residents aged ≥ 60 years were recruited from May 2021 to September 2021 and had a comprehensive geriatric evaluation. The diagnosis of sarcopenia followed the criteria issued by the Asian Working Group for Sarcopenia in 2019. MCR was defined as subjective cognitive decline and slow gait. Apathy symptoms and physical activity were assessed by the Apathy Evaluation Scale (AES) and the International Physical Activity Questionnaire (IPAQ). Logistic regression and moderated mediation analyses were conducted to explore the association between the four.

**Results:**

60 (7.1%) had MCR among 846 participants. After full adjustment, sarcopenia (odds ratio [OR] = 3.81, 95% confidence interval [CI] = 1.69–8.60, *P* = 0.001), AES score (OR = 1.09, 95% CI = 1.04–1.14, *P* < 0.001), and IPAQ level (OR = 0.43, 95% CI = 0.28–0.66, *P* < 0.001) were associated with MCR. Apathy partially mediated the relationship between sarcopenia and MCR. Physical activity played a moderation role in the indirect pathway of the mediation model. The increase in physical activity can alleviate the indirect effect of sarcopenia on MCR.

**Conclusion:**

We established a moderated mediation model to uncover the underlying association mechanism of sarcopenia and MCR preliminarily. These findings suggest that attention should be paid to the management of apathy and physical activity in the context of sarcopenia to prevent early dementia actively. Further validation is needed in future longitudinal studies.

## Introduction

Motoric cognitive risk syndrome (MCR) was proposed and verified in 2013, defined as cognitive complaints and slow gait in the elderly without dementia and mobility disability [1]. Slow gait and cognitive complaints have been proven to be the early risk markers for dementia [2, 3], based on which MCR was established. Both MCR and mild cognitive impairment (MCI) are the intermediate state between normal cognitive aging and dementia [1]. Compared with MCI, the diagnosis of MCR substitutes the objective cognitive impairment criterion with a slow gait, which effectively complements the identification of individuals at high risk of dementia. Even after accounting for diagnostic overlap with MCI subtypes, MCR is related to future dementia, especially vascular dementia [1]. In addition, although MCR is a motor-based syndrome, the ability to predict the risk of dementia transformation once diagnosed with MCR is derived from the degree of cognitive impairment rather than motor disorder [4]. Prospective studies have shown that MCR is an independent risk factor for dementia [1, 4, 5] and death [6].

Sarcopenia is an age-related loss in skeletal muscle mass accompanied by decreased muscle strength or physical performance [7]. Recent studies have shown that, except for influence on physical function, sarcopenia is related to cognitive impairment in the elderly [8, 9]. Beeri et al. [10] reported sarcopenia as predictors of late-life Alzheimer’s disease (AD), MCI, and cognitive decline in older adults. Diagnostic components of sarcopenia (muscle mass [11, 12], muscle strength [13], and physical function [13, 14]) were also independently associated with cognitive impairment to varying degrees. Moreover, among people who already had cognitive impairment, Sugimoto et al. [15] found an increased prevalence of sarcopenia from amnestic MCI to AD. However, these researches focused on dementia and MCI. No study to date has explored the association of MCR with sarcopenia. Whether sarcopenia is involved in the pathological mechanism of MCR remains an open issue.

Traditionally, apathy is a neuropsychiatric syndrome of primary motivational loss, which cannot attribute to emotional disturbance, intellectual damage, or attenuate consciousness [16]. Lack of motivation is the core symptom, evidenced by the quantitative reduction in goal-directed behaviors [17]. Apathy was once considered a sub-symptom of depression, but subsequent studies have found the difference in the disease course, clinical symptoms, and treatment methods [18, 19]. Apathy is highly prevalent in many neurological disorders and is associated with cognitive impairment in these patients [20–22]. Meanwhile, longitudinal studies documented that apathy also correlated to cognitive decline [23] and increased risk of dementia [24] among the community elderly. Apathy predicted incident MCR in community older adults [25]. On the other hand, handgrip strength and other sarcopenia-related physical performances were associated with affective functions of subjects from the specialized dementia outpatient clinic, such as apathy [26]. Considering its association with sarcopenia and MCR respective, it is suspected that apathy may be a mediating factor between the two.

Physical activity affects physical and cognitive function. Inactivity contributes to the development of sarcopenia [27]. Additionally, decreased daytime motor activity was found in AD patients with apathy [28], and low vitality was considered as the dementia-specific risk factor for sarcopenia [15]. In contrast, physical exercise improved sarcopenia status [29], and positively affected cognitive function in both older adults with and without cognitive impairment [30, 31]. These studies suggest that physical activity may moderate the relationship between sarcopenia and cognitive impairment.

Current treatments for dementia are often unsatisfactory, so there is a need to focus on the early stages of cognitive impairment to develop effective prevention strategies. As a pre-dementia syndrome, MCR is reversible before further cognitive decline [32]. Sarcopenia is also an invertible geriatric syndrome. Resistance training and amino acid supplementation can effectively improve sarcopenia status [29, 33, 34]. Therefore, exploring the relationship between sarcopenia, apathy, and MCR may provide a basis for effective intervention strategies before clinical adverse events. This study hypothesized that sarcopenia correlated to MCR, in which apathy played a mediating role and physical activity worked as a moderator.

## Methods

### Study population

We invited community residents, who were ≥ 60 years and independent in basic daily life, to participate in the study from May 2021 to September 2021 in Xuzhou, China. The final analytic sample consisted of 846 participants after excluding 101 subjects. The exclusions were as follows: 17 participants failed to measure body composition analysis due to cardiac stents or pacemakers; 6 participants were unable to perform the physical function tests due to severe lumbar disc herniation or hip disease; 78 participants were lack of covariates. No participants in this study had dementia, Parkinson's disease, multiple sclerosis, and hemiplegia. All participants provided informed consent. The Ethics Committee of the Affiliated Hospital of Xuzhou Medical University approved this research, and the study methods followed the principles of the Declaration of Helsinki.

### Definition of MCR

The operational definition of MCR is the presence of subjective cognitive decline (SCD) and slow gait in subjects without dementia and mobility disability. The diagnostic procedure is as follows [1, 35]:

1. SCD is determined based on the positive response to a memory item in the 15-item Geriatric Depression Scale (GDS) [36], whose standardized question is "Do you feel you have more problems with memory than most?".

2. Slow gait is defined as the usual walking speed one standard deviation or more below age- and gender-specific mean values established in the study population (Table 1).

**Table 1:** The gait speed cut-off values of slow gait for Motoric Cognitive Risk syndrome

Age group (y)	Male (m/s)	Female (m/s)
60–64	1.064	0.935
65–69	0.872	0.845
70–74	0.814	0.781
75–79	0.775	0.729
80–84	0.715	0.585
85 +	0.610	0.512

3. Absence of mobility disability refers to no difficulty in dressing, eating, bathing, going to the toilet, continence, and transferring [37].

4. There is no confirmed dementia. In addition, the Clinical Dementia Rating Scale (CDRS) score is less than or equal to 0.5 [38].

### Diagnosis of sarcopenia

Based on the diagnostic criteria issued by the Asian Working Group of Sarcopenia (AWGS) in 2019, the definition of sarcopenia is low muscle mass plus low muscle strength or poor physical performance [39]. Muscle mass was measured by direct segmental multi-frequency bioelectrical impedance analysis (InBody270; Biospace Co., Ltd, Seoul, Korea). The skeletal muscle index (SMI) is the relative skeletal muscle mass of limbs divided by the square of height. Low muscle mass means SMI is lower than 7.0 kg/m^2^ in men or 5.7 kg/m^2^ in women.

Muscle strength was evaluated using a handheld dynamometer (EH101; Xiangshan Inc., Guangdong, China). Participants were asked to use their dominant hands to exert the best effort twice, recording the maximum value for analysis. Low muscle strength refers to handgrip strength < 28 kg in men or < 18 kg in women.

AWGS 2019 recommends defining low physical performance based on the Short-Physical Performance Battery (SPPB), or 6-m gait speed test, or five-times sit-to-stand test (FTSST). The diagnosis of MCR already contains a slow gait. To avoid the overlapping effect of gait speed on the relationship between sarcopenia and MCR, we used FTSST without walking speed test as the index of physical function in sarcopenia. Participants were asked to stand up from a standard armless chair five times as fast as possible, crossing their upper limbs upon the chest. They were instructed to stand up with their legs fully straightened and sit down firmly in each sit-to-stand process. The FTSST time ≥ 12 s is the cut-off value for poor physical performance.

### Assessment of apathy symptoms

The Apathy Evaluation Scale (AES) self-rated version was used to quantify apathy symptoms [40]. The AES consists of 18 items scored on a 4-point Likert type scale, representing one of the most clinically used scales for apathy. It identifies apathy based on three areas: emotional responsiveness, thought content, and observation of activity. 37 is the cut-off score for apathy [41]. The higher the score, the more serious the apathy symptoms.

### Evaluation of physical activity

We evaluated physical activity by the International Physical Activity Questionnaire (IPAQ) [42]. The scale assesses the amount of time spent on vigorous activity, moderate-intensity activity, walking, and sitting during the previous seven days. Metabolic Equivalent of Energy (MET) values were assigned according to activity intensity. Physical activity levels were classified as low, medium, and high.

### Other measurements

We collected basic information through a standard questionnaire, including age, gender, education level, night sleep duration, the number of prescription drugs, chronic medical conditions, smoking history, drinking history, fall history last year, self-perceived vision status, and self-perceived health status. Chronic diseases include hypertension, dyslipidemia, diabetes, gout, coronary heart disease, asthma, chronic respiratory diseases, arthritis, osteoporosis, peptic ulcer, thyroid disease, cancer. Smoking/drinking refers to smoking/drinking daily for more than three months. Self-perceived visual/health status was to ask participants how to view their own visual/health. Ratings included poor, fair, good, and excellent, among which "poor" and "fair" meant poor self-perceived visual/health. We also measured the height and weight on-site to calculate body mass index (BMI) (weight divided by height squared). The Chinese version of the mini-mental state examination (MMSE) was used to evaluate cognitive function [43]. Nutritional status was assessed by the Mini Nutritional Assessment Short-Form (MNA-SF) [44]. The 15-item GDS was used to assess depressive symptoms [36].

### Statistical analyses

Firstly, we compared characteristics between non-MCR and MCR participants. Continuous variables are expressed as mean ± SD or median (25–75 percentiles) as appropriate. Categorical variables are expressed as proportions. The independent t-test or Wilcoxon rank-sum test was used for continuous variables, and the chi-square test was used for categorical variables. Secondly, univariate and multivariate logistic regression analyses were conducted to explore the association of MCR with sarcopenia, apathy, and physical activity. Finally, we estimated the moderated mediation model of sarcopenia and MCR using Mplus software. Mplus can build structural equation modelling (SEM) and allows dichotomous outcome variables in the model [45, 46]. The Mplus codes for mediation models are available at the website [47]. Maximum-likelihood estimation in Mplus conducts the default logistic regression for a binary outcome variable and requires numerical integration. Therefore, the direct and indirect effects of SEM are defined based on a continuous latent response variable underlying the binary outcome [45]. Covariates included gender, age, education level, BMI, and health-related variables with statistical differences between groups. All continuous variables were centered using the group mean before the model fit to facilitate the interpretation of the results. We set 5000 repeated bootstrap samples to calculate the bias-corrected 95% confidence interval. The coefficient of the path analysis is statistically significant if the confidence interval does not contain zero, while the effect in an odds ratio metric is statistically significant if the confidence interval does not cover one. All statistical analyses were performed using IBM SPSS Statistics for Windows, version 25.0 (SPSS Inc, Chicago, Illinois, the United States) and Mplus version 8.3 (Muthén & Muthén, Los Angeles, California, the United States). Statistical significance was a two-sided *P* < 0.05.

## Results

### Characteristics for MCR

Among the 846 participants, 60 (7.1%) had MCR, including 40 (7.9%) females and 20 (5.9%) males. Table 2 presents the characteristics of the study population grouped by MCR. Individuals with MCR tended to be older and less educated (*P* < 0.05), whereas there was no difference in gender and BMI between groups. In health-related variables, night sleep duration, number of prescription drugs, number of chronic diseases, chronic respiratory diseases, arthritis, MNA-SF, IPAQ, fall history last year, poor self-perceived vision, poor self-perceived health, GDS score, AES score, MMSE score, sarcopenia, SMI, handgrip strength, FTSST time and gait speed were significantly different between groups (*P* < 0.05).

**Table 2:** Characteristics of study participants according to Motoric Cognitive Risk syndrome status

Characteristic	Non-MCR (*n* = 786)	MCR (*n* = 60)	*P* value
Age (years)	71.0 ± 8.1	74.4 ± 8.6	0.002
Gender			
Male (%)	318 (40.5)	20 (33.3)	
Female (%)	468 (59.5)	40 (66.7)	0.277
BMI (kg/m^2^)	24.39 ± 3.19	24.11 ± 3.72	0.516
Education level (%)			
Illiteracy	111 (14.1)	19 (31.7)	
Primary school	147 (18.7)	14 (23.3)	
Secondary school	445 (56.6)	26 (43.3)	
College	83 (10.6)	1 (1.7)	0.001
Night sleep duration (h)	6.4 ± 0.9	6.0 ± 1.4	0.033
Smoking (%)	118 (15.0)	8 (13.3)	0.725
Drinking (%)	107 (13.6)	4 (6.7)	0.124
Number of prescription drugs	1.0 (0.0, 2.0)	1.5 (0.0, 4.0)	0.001
Number of chronic diseases	1.0 (0.0, 2.0)	1.5 (0.5, 2.5)	0.033
Chronic conditions (%)			
chronic respiratory diseases	29 (3.7)	9 (15.0)	< 0.001
Hypertension	300 (38.2)	23 (38.3)	0.980
diabetes	113 (14.4)	12 (20.0)	0.237
Arthritis	158 (20.1)	19 (31.7)	0.034
Osteoporosis	65 (8.3)	2 (3.3)	0.264
MNA-SF score	14 (13, 14)	13 (12, 14)	0.001
IPAQ (Met/week)	2398 (1716, 2982)	1704 (940, 2166)	< 0.001
IPAQ level			
Low	15 (1.9)	11 (18.3)	
Medium	575 (73.2)	44 (73.3)	
High	196 (24.9)	5 (8.3)	< 0.001
Fall history last year	55 (7.0)	14 (23.3)	< 0.001
Self-perceived vision status	488 (62.1)	50 (83.3)	0.001
Self-perceived health status	248 (31.6)	33 (55.0)	< 0.001
GDS score	1 (0, 2)	3 (2, 6)	< 0.001
AES score	29.5 ± 6.9	38.8 ± 9.2	< 0.001
MMSE score	27 (25, 29)	24 (21, 27)	< 0.001
Sarcopenia (%)	76 (9.7)	21 (35.0)	< 0.001
SMI (kg/m^2^)	6.79 ± 0.99	6.37 ± 0.86	< 0.001
Handgrip strength (kg)	27.23 ± 8.94	20.22 ± 6.66	< 0.001
FTSST time (s)	10.60 ± 3.04	15.21 ± 3.92	< 0.001
Gait speed (m/s)	1.05 ± 0.24	0.65 ± 0.15	< 0.001

*BMI* body mass index, *MNA-SF* Mini Nutritional Assessment Short-Form, *IPAQ* International Physical Activity Questionnaire, Met/week metabolic equivalent task minutes per week, *GDS* Geriatric Depression Scale, *AES* Apathy Evaluation Scale, *MMSE* mini-mental state examination, *SMI* skeletal muscle index, *FTSST* five-times sit-to-stand test

### Associations of sarcopenia, apathy, and physical activity with MCR

Sarcopenia, apathy, and physical activity were associated with MCR both in univariate and multivariate logistic regression models (Table 3). After adjustment for all covariates, sarcopenia (odds ratio [OR] = 3.81, 95% confidence interval [CI] = 1.69–8.60, *P* = 0.001) and AES score (OR = 1.09, 95% CI = 1.04–1.14, *P* < 0.001) were positively related to MCR. Physical activity (OR = 0.43, 95% CI = 0.28–0.66, *P* < 0.001) was negatively associated with MCR.

**Table 3:** Associations of sarcopenia, apathy, and physical activity with MCR

Variables	Unadjusted model		Adjusted model 1		Adjusted model 2	
	OR (95% Cl)	*P* value	OR (95% Cl)	*P* value	OR (95% Cl)	*P* value
Sarcopenia	5.03(2.81, 8.99)	< 0.001	3.10(1.46, 6.56)	0.003	3.81(1.69, 8.60)	0.001
AES score	1.15(1.11, 1.19)	< 0.001	1.09(1.04, 1.14)	< 0.001	1.09(1.04, 1.14)	< 0.001
IPAQ	0.32(0.22, 0.47)	< 0.001	0.42(0.28, 0.64)	< 0.001	0.43(0.28, 0.66)	< 0.001

*SMI* skeletal muscle index, *FTSST* five-times sit-to-stand test, *IPAQ* International Physical Activity Questionnaire, *AES* Apathy Evaluation Scale, *CI* confidence interval. Adjusted model 1 had adjusted potential confounders including age; education level; night sleep duration; Mini Nutritional Assessment Short-Form score; the number of prescription drugs; the number of chronic diseases; chronic respiratory diseases; Arthritis; Geriatric Depression Scale score; self-perceived vision status; self-perceived health status; fall history last year. Adjusted model 2 had adjusted all the covariates (model 2 plus gender; body mass index; smoking; drinking; hypertension; diabetes; osteoporosis)

### Mediation model

**Fig. 1:**
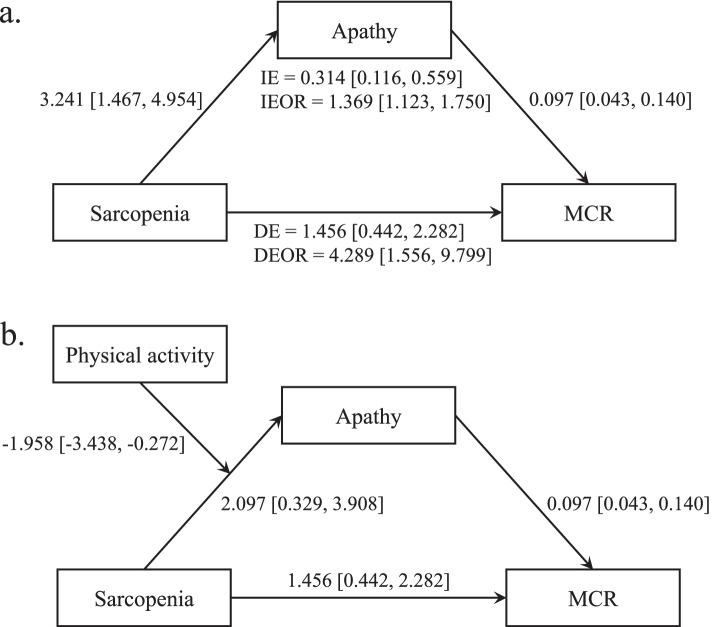
Mediation and moderated mediation path diagram. Panel a, the mediating effect of apathy on the relationship between sarcopenia and MCR. Panel b, the moderating effect of physical activity on the mediation model. The effect values and their corresponding confidence intervals between variables are marked next to the arrow. DE, direct effect; IE, Indirect effect; DEOR/IEOR, direct/indirect effect odds ratio

Path analysis showed that the pathway of sarcopenia to apathy, apathy to MCR, and sarcopenia to MCR were statistically significant (all *P* < 0.01). The point estimate of the indirect effect was 0.314 (bootstrap estimated 95% CI = 0.116–0.559, *P* = 0.007). The indirect effect odds ratio was 1.369 (bootstrap estimated 95% CI = 1.123–1.750, *P* < 0.001), which meant that the odds ratio of MCR prevalence increased by 0.369 due to the mediational mechanism of apathy. The direct effect and the direct effect odds ratio of sarcopenia on MCR were 1.456 (bootstrap estimated 95% CI = 0.442–2.282, *P* = 0.002) and 4.289 (bootstrap estimated 95% CI = 1.556–9.799, *P* = 0.121), respectively (Table 4, Fig. 1a). This indicated that apathy partially mediated the association between sarcopenia and MCR. The ratio of the indirect effect odds ratio to the direct effect odds ratio is 31.92%.

**Table 4:** Path analysis of the mediation and moderated mediation model using structural equation modelling

Dependent variable	Independent variable	Effect	Estimate	SE	Est/SE	*P* value	BootLLCI	BootLLCI
Mediation model								
Apathy	Sarcopenia		3.241	0.896	3.615	< 0.001	1.467	4.954
MCR	Sarcopenia		1.456	0.476	3.061	0.002	0.442	2.282
	Apathy		0.097	0.025	3.813	< 0.001	0.043	0.140
		Direct effect	1.456	0.476	3.061	0.002	0.442	2.282
		Direct effect odds ratio	4.289	2.770	1.549	0.121	1.556	9.799
		Indirect effect	0.314	0.117	2.685	0.007	0.116	0.559
		Indirect effect odds ratio	1.369	0.170	8.031	< 0.001	1.123	1.750
Moderated mediation model								
Apathy	Sarcopenia		2.097	0.926	2.265	0.024	0.329	3.908
	Physical activity		-1.234	0.276	-4.467	< 0.001	-1.766	-0.673
	Sarcopenia*Physical activity		-1.958	0.791	-2.475	0.013	-3.438	-0.272
MCR	Sarcopenia		1.456	0.476	3.061	0.002	0.442	2.282
	Apathy		0.097	0.025	3.813	< 0.001	0.043	0.140

*MCR* motoric cognitive risk syndrome, *SE* standard error, *Est/SE* estimate divided by the standard error. BootLLCI / BootLLCI, lower/upper 2.5% of bias-corrected confidence interval derived from bootstrap estimates. Adjusted for gender, body mass index, and all potential confounding factors

### Moderated mediation model

We first tested the moderation effects of physical activity on all of the sarcopenia-apathy path, the apathy-MCR path, and the direct sarcopenia-MCR path. The result revealed that physical activity only significantly moderated the sarcopenia-apathy path. Therefore, we refit the model to examine the moderation effect of physical activity on the first half of the mediation path. As shown in the Table 4 and Fig. 1b, the point estimate of moderating effect was -1.958 (bootstrap estimated 95% CI = -3.438–-0.272, *P* = 0.013). This suggested that physical activity moderated the indirect pathways of sarcopenia to MCR.

We further analyzed the conditional indirect effects of sarcopenia on MCR under different levels of physical activity. From low to medium physical activity level, the indirect effects (low: β = 0.393, bootstrap estimated 95% CI = 0.161–0.696; medium: β = 0.203, bootstrap estimated 95% CI = 0.026–0.428) and indirect effects odds ratio (low: OR = 1.482, bootstrap estimated 95% CI = 1.175–2.006; medium: OR = 1.225, bootstrap estimated 95% CI = 1.027–1.534) were significant, and the effect size decreased with increasing physical activity level. When the physical activity was high, the indirect effects (β = 0.013, bootstrap estimated 95% CI = -0.285–0.309) and indirect effects odds ratio (OR = 1.014, bootstrap estimated 95% CI = 0.752–1.362) of sarcopenia on MCR were both insignificant. Whether in an odds ratio metric or not, the differences in indirect effects between the three groups were all statistically significant (Table 5). It could be conceived that physical activity alleviated the indirect effect of sarcopenia on MCR, and the indirect effect became in-exist under the high physical activity level.

**Table 5:** The conditional indirect effects under different levels of physical activity

Indirect effect scale	Physical activity level	Comparison between levels	Estimate	SE	BootLLCI	BootLLCI
β	Low		0.393^a^	0.141	0.161	0.696
	Medium		0.203^a^	0.104	0.026	0.428
	High		0.013	0.148	-0.285	0.309
		Medium vs Low	-0.190^a^	0.100	-0.400	-0.018
		High vs Medium	-0.190^a^	0.100	-0.400	-0.018
		High vs Low	-0.380^a^	0.199	-0.801	-0.036
OR	Low		1.482^a^	0.227	1.175	2.006
	Medium		1.225^a^	0.133	1.027	1.534
	High		1.014	0.152	0.752	1.362
		Medium vs Low	-0.256^a^	0.163	-0.628	-0.022
		High vs Medium	-0.212^a^	0.104	-0.425	-0.022
		High vs Low	-0.468^a^	0.266	-1.054	-0.044

*SE* standard error. BootLLCI / BootLLCI, lower/upper 2.5% of bias-corrected confidence interval derived from bootstrap estimates. The low, medium, and high values of physical activity referred to 1 SD below mean, mean, and 1 SD above mean, respectively. ^a^The effect or effect difference was statistically significant

## Discussion

The study examined the association between sarcopenia and MCR, and further revealed the underlying mechanisms of apathy and physical activity in the association by establishing a moderated mediation model. We found a positive connection between sarcopenia and MCR. Apathy partially mediated the effect of sarcopenia on MCR, and physical activity moderated the indirect pathway in the mediation model. The results supported the possibility of modifying the effects of sarcopenia on MCR by altering apathy and physical activity.

A systematic review of more than 20,000 older adults in 17 countries reported the prevalence of MCR ranging from 5.3% to 15.5%, with a pooled prevalence of 9.7% [48]. The prevalence of MCR in this study was 7.1%, somewhat lower than that previously reported. One possible reason is that our diagnosis of SCD was relatively strict. Older people were considered to have SCD only when they felt they had more memory problems than most people. By contrast, participants were diagnosed with SCD when their self-perceived memory was either poor or worse than before in other researches [6, 49]. An alternative explanation is that our study participants were relatively healthy community residents rather than outpatients or inpatients, which may underestimate the prevalence of MCR.

As far as we know, our study is the first to identify the relationship between sarcopenia and MCR. The results showed that sarcopenia was independently associated with a nearly threefold increased risk of MCR in the community elderly. There has been no comparable research to date on the relationship between sarcopenia and MCR. Nevertheless, the associations of sarcopenia with cognitive impairment and dementia have been controversially reported. A recent meta-analysis reported that sarcopenia was related to cognitive impairment independent of their different operational definitions and study population [9]. However, the exact mechanisms involved have not been determined. The association between sarcopenia and MCR in this study seemed to be partly explained from the perspective of physical function. Although we chose FTSST instead of the usual gait speed test in the diagnosis of sarcopenia, gait speed was significantly lower in participants with sarcopenia than normal individuals (0.76 m/s vs. 1.06 m/s, *P* < 0.001). Meanwhile, slow gait is also a motor manifestation of MCR. Thus, the link between sarcopenia and MCR may be driven by slow gait to some extent. Besides, the association between sarcopenia and motoric cognitive impairment further supported sarcopenia as a contributing factor to cognitive decline.

The mediation analysis showed that apathy was positively related to sarcopenia and MCR, and partially mediated the effect of sarcopenia on MCR. Ceide et al. [25] reported that apathy predicted future MCR but not MCI in community older adults. They also found GDS score became MCR-independent after removing the 3-item subscale of apathy in GDS [25]. A prospective study showed that apathy rather than depression was most strongly related to subsequent weight loss in AD patients [50]. Several common underlying risk factors may explain the serial relationships. Firstly, malnutrition is a recognized risk factor for sarcopenia [51] and cognitive impairment [52] in older adults. Among older adults with mild cognitive decline, nutritional status was also independently associated with apathy [53]. In this study, The MNA-SF score of participants with MCR or sarcopenia or apathy was significantly lower than that of normal individuals (all *P* < 0.001). Secondly, there may be hormonal dysregulation. Insulin is involved in skeletal muscle metabolism as a synthetic metabolic hormone [54]. Besides, abnormal glucose metabolism caused by insulin resistance is considered a vital pathological mechanism of AD [55]. Burns et al. [56, 57] found that increased peripheral insulin was related to reduced lean mass, cognitive dysfunction, and brain atrophy in early AD patients. Thirdly, age-related inflammatory abnormalities can be a shared underlying factor. A longitudinal cohort study found that interleukin 6 and C-reactive protein (CRP) contributed to appendicular skeletal muscle loss in healthy individuals[58]. Meanwhile, Sathyan et al. [59] prospectively clarified that the overexpression of interleukin 10 was associated with incident MCR. A population-based cohort study found increased CRP levels remained associated with apathy symptoms after adjustment for demographics and depressive symptoms [60].

A moderated mediation model showed that physical activity moderated the association between sarcopenia and apathy. The indirect effect of sarcopenia on MCR weakened as physical activity levels increased. Physical inactivity is a motor characteristic of sarcopenia and apathy. It can also lead to cerebral hypoperfusion resulting in cognitive impairment [61]. In addition, myokines molecules secreted by skeletal muscles during exercise can regulate cerebral functions, including emotion and cognition [62]. Therefore, physical exercise supported the existence of muscle-brain cross-talk [63]. Our study suggested that physical exercise could buffer or even counteract the adverse effects of sarcopenia on apathy. Therefore, increasing physical activity is not only an essential means to improve sarcopenia status and apathy symptoms but also an effective strategy to prevent sarcopenia from promoting early dementia. Future research could investigate whether physical activity improves cognitive function in patients with sarcopenia.

There are some limitations to this study. First, the research only preliminarily discussed the mechanism of sarcopenia on MCR based on a cross-sectional design. The reverse connection between MCR and sarcopenia can also exist. Future longitudinal studies will provide strong evidence of causality. Second, the total effect is not exactly the sum of the indirect effect and the direct effect in the traditional mediation analysis with a binary outcome variable, so we did not directly compare the indirect effect with the total effect. Additionally, it could not be ruled out other potential mediators that have not yet been explored. Third, some key variables in this study relied heavily on the participants’ self-report, such as apathy, physical activity, and subjective cognitive decline. We admitted that it was difficult to evaluate these latent variables with objective methods. Participants may have recall bias and exaggeration or concealment in their statements, leading to deviations between the measurements and the actual situation. Finally, the distribution of our sample population was relatively limited, which required multi-center studies for further verification. However, we believed that the sample size of this study was enough by calculating the statistical power. The univariate logistic regression of MCR on sarcopenia with a sample size of 846 observations (of which 89% were non-sarcopenia and 11% were sarcopenia) achieved 95% power at a significance level of 0.05 and an odds ratio of 5.023.

## Conclusions

Our work initially investigated the association and potential mechanism between sarcopenia and MCR. Sarcopenia was positively correlated with MCR. Sarcopenia can promote MCR through apathy symptoms, while physical activity moderated this association to some extent. Exploring these mediation pathways can provide theoretical guidance for early detection and intervention of at-risk individuals in the community elderly. At the same time, the effect mechanism of apathy and physical activity emphasizes the prevention of early dementia in the context of combining physical and mental management, such as the promotion of physical activity, nutritional support, psycho-behavioral therapy (e.g., development of interests, horticultural activity). Further longitudinal studies and intervention studies are needed to clarify the mechanisms involved.

## Data Availability

The datasets used and/or analysed during the current study are available from the corresponding author on reasonable request.

## Abbreviations

MCR: Motoric cognitive risk
AES: Apathy evaluation scale
IPAQ: International physical activity questionnaire
OR: Odds ratio
CI: Confidence interval
MCI: Mild cognitive impairment
AD: Alzheimer’s disease
SCD: Subjective cognitive decline
CDRS: Clinical dementia rating scale
AWGS: Asian Working Group of Sarcopenia
SMI: Skeletal muscle index
FTSST: Five-times sit-to-stand test
MET: Metabolic equivalent of energy
BMI: Body mass index
MMSE: Mini-mental state examination
MNA-SF: Mini nutritional assessment short-form
GDS: Geriatric depression scale
SEM: Structural equation modelling
CRP: C-reactive protein
